# A Case Report: Electrotonic Modulation‐Related T‐Wave Over‐Sensing After Left Bundle Branch Pacing

**DOI:** 10.1111/anec.70083

**Published:** 2025-04-28

**Authors:** Linlin Li, Manxin Lin, Jincun Guo, Qiang Li, Fanqi Meng, Xinyi Huang, Simei Chen, Binni Cai

**Affiliations:** ^1^ Division of Cardiology, Xiamen Cardiovascular Hospital Xiamen University Xiamen Fujian China; ^2^ Division of Echocardiography, Xiamen Cardiovascular Hospital Xiamen University Xiamen Fujian China; ^3^ Division of Cardiac Function, Xiamen Cardiovascular Hospital Xiamen University Xiamen Fujian China

**Keywords:** cardiac resynchronization therapy (CRT), left bundle branch pacing, T‐wave oversensing

## Abstract

A 66‐year‐old male patient diagnosed with dilated cardiomyopathy, heart failure with reduced EF (32%), and complete left bundle branch block (CLBBB) received cardiac resynchronization therapy (CRT)‐D implantation. Left bundle branch pacing (LBBP) was successfully performed, but during the follow‐up 6 weeks later, the electrocardiogram (ECG) showed a sinus rhythm tracked by ventricular pacing with a ratio of approximately 2:1 due to T‐wave over‐sensing, which might be caused by the changes in T‐wave morphology due to electrotonic modulation and hyperkalemia or by the lower sensitivity threshold set by the auto sensing algorithm of the ICD. Shortening post‐ventricular atrial refractory period (PVARP) restored the ventricular pacing tracking of the atrium, and the T‐wave changes improved as time went by.

## Graphical abstract

A 66‐year‐old male patient received successful left bundle branch pacing, but the follow‐up electrocardiogram (ECG) showed a sinus rhythm tracked by ventricular pacing with a ratio of approximately 2:1 due to T‐wave over‐sensing, which affected the efficacy of cardiac resynchronization therapy (CRT). Shortening post‐ventricular atrial refractory period (PVARP) restored the ventricular pacing tracking of the atrium, and the T‐wave changes improved as time went by.

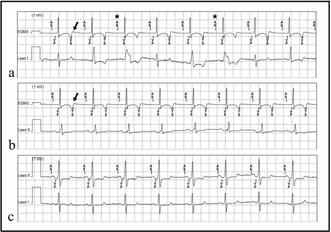

## 1 Case Report

**FIGURE 1:**
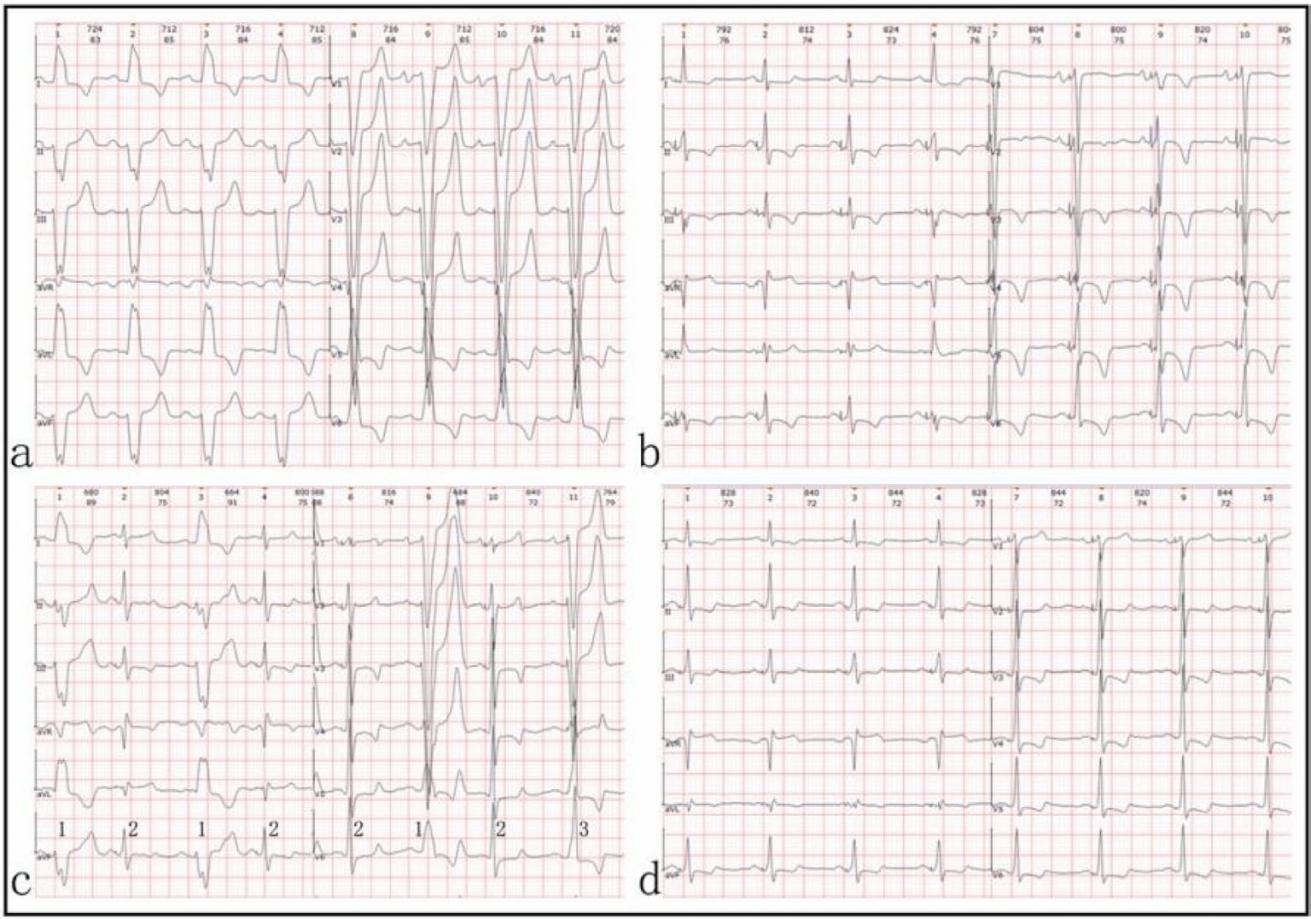
Surface electrocardiograms (ECG) of the patient. (a) ECG of the patient before the procedure. Sinus rhythm, CLBBB, which was according to the criteria of Strauss. The duration of the QRS complex was 165 ms, and the QT interval was 460 ms. QTc was 541 ms. (b) ECG after the procedure with optimal programming. Narrow QRS complex (97 ms) with significant T‐wave inversion. QTc was 429 ms. (c) ECG 6 weeks after implantation. Sinus rhythm (80 bpm), tracked by ventricular pacing with a ratio of approximately 2:1. c‐1 intrinsic QRS complex. c‐2 QRS under LBBP with optimal programming. c‐3 a fusion of pacing and intrinsic conduction. QTc under pacing was 503 ms. (d) ECG 12 weeks after implantation. Sinus rhythm, tracked by ventricular pacing with a ratio of 1:1. QT interval was 402 ms. QTc was 421 ms.

A 66‐year‐old male patient presented with dyspnea on exertion had been admitted to the hospital repeatedly for 4 years. The patient had a past medical history of chronic kidney failure (Scr: 286 μmol/L upon admission). Only mild stenosis was found in coronary angiography. Electrocardiogram (ECG) manifested a wide QRS complex with CLBBB pattern (Figure 1a, QRS 164 ms), which was accordant with the diagnostic criteria of true CLBBB proposed by Strauss et al. (2011). Echocardiography showed an increased left ventricular diastolic diameter (LVDd) of 60 mm and a reduced ejection fraction (EF) of 32%. He was diagnosed with dilated cardiomyopathy (NYHA III) and had received optimized medication for more than 1 year without any improvement, at which point cardiac resynchronization therapy was indicated. Thus, the patient received CRT‐D implantation in our center.

## 2 Implantation Procedure

**FIGURE 2:**
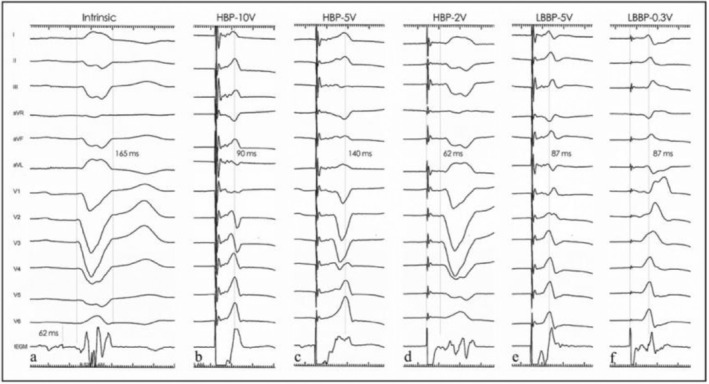
Intracardiac electrograms (EGM) during the procedure. (a) EGM under intrinsic conduction. His potential was recorded. HV interval was 62 ms. (b–d) EGM under HBP with different outputs, from 10 V to 2 V. The CLBBB was corrected completely when pacing under 10 V/0.5 ms, partially corrected under 5v/0.5 ms, and failed in correcting the LBBB under 2v/0.5 ms. (e, f) EGM under LBBP with high and low output, respectively, shared the same stim‐LVAT (87 ms). When pacing under the threshold output (0.3v/0.5 ms), the QRS morphology presented an rSR pattern in V1, with an equipotential line between the stimulus and the QRS complex, which was identified as a selective LBBP pattern.

The defibrillation lead was implanted into the apical area of the right ventricle; afterwards, the pacing lead (model 3830, 69 cm, Medtronic Inc.) was delivered through a fixed‐curve sheath (C315 His, Medtronic Inc.) into the His bundle area. Electrogram under intrinsic conduction was then recorded, showing His potential with an HV interval of 62 ms (Figure 2a). LBBB could be corrected completely when pacing under 10 V/0.5 ms at this position, partially corrected under 5v/0.5 ms and failed in correcting the LBBB under 2v/0.5 ms (Figure 2b–d). Another 3830 lead was positioned about 1.0 cm away from the primary lead and headed to the apex, where the pacing QRS morphology presented a “W” type in the V1 lead. The pacing lead was screwed under careful monitoring until the pacing QRS presented a QR morphology and the stimulus to left ventricular activation time (Sti‐LVAT) stayed stable (87 ms) under both high and low output (Figure 2e,f). The first lead (3830) for HBP was then withdrawn and implanted into the right atrium septal, and CRT‐D (D364TRG, Medtronic Inc.) was applied.

## 3 Follow‐Up

**FIGURE 3:**
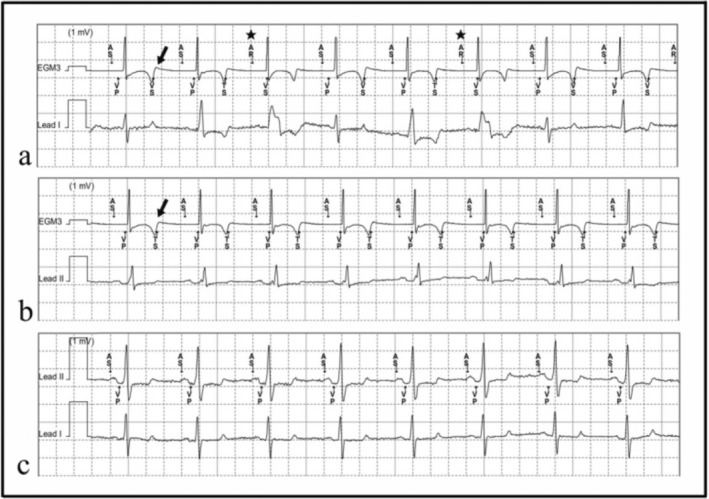
Device Intracardiac electrograms after procedure. (a, b) EGM before and after reprogramming 6 weeks after procedure. (a) There are three different patterns of QRS complexes that appear in “trigeminy” from Lead I: narrow, intermediate, and widened. The first narrow QRS complex was the optimal result of LBBP merging with intrinsic right bundle branch conduction, but the following TWO resulted in the pacemaker reaching its upper tracking rate limit and triggered the prolongation of SAVD, which in turn led to an intermediate fusion beat formed by the improper merging of intrinsic and paced QRS. Moreover, the progressive prolongation of SAVD resulted in the delay of QRS complex and subsequent TWO, causing the next intrinsic atrial signal to fall into PVARP and fail to trigger ventricular pacing, which led to the widened baseline CLBBB QRS morphology. (b) PVARP was shortened, resulting in the P wave falling outside the PVARP and identified correctly as atrial sense (AS), making ventricular tracking of the atrial signal possible with a ratio of 1:1. Upper tracking rate limit was reset higher as well. (c) EGM 12 weeks after procedure. TWOs disappeared.

The patient received optimal program and medication therapy including irbesartan, metoprolol, and spironolactone before discharge. When the sensed atrioventricular delay (SAVD) was set to 100 ms, the pacing QRS complex presented a full normalization with ST‐T changes and a QT interval of 429 ms (Figure 1b). Six weeks after the CRT‐D implantation, the patient came back for a routine follow‐up without any discomfort. Upon physical examination, the heart rhythm was found to be regular, but the heart sound alternated from strong to weak beat to beat. ECG showed a sinus rhythm tracked by ventricular pacing with a ratio of approximately 2:1 (Figure 1c). Interrogation of the pacemaker revealed T‐wave over‐sensing (TWO) after ventricular pacing, which resulted in some P waves falling in the post‐ventricular atrial refractory period (PVARP) and failing to trigger the following ventricular pacing (Figure 3a). The blood test showed hyperkalemia (6.1 mmol/L). The level of potassium ion decreased to normal (5.2 mmol/L) after treatment, and the pacemaker was reprogrammed by changing the ventricular sensing polarity and reducing the ventricular sensitivity. However, the TWO could not be eliminated with these methods. Therefore, PVARP was shortened during pacemaker programming to restore ventricular pacing tracking of the atrial signal with a ratio of 1:1 (Figure 3b). During the next follow‐up 3 months later, the patient's heart function improved to NYHA I, with ECG showing sinus rhythm, alleviated T‐wave inversion, and a QT interval of 402 ms (Figure 1d). Device intracardiac electrogram (IEGM) revealed no T‐wave over‐sensing (Figure 3c). The chest X‐ray showed a remarkable reversion of cardiomegaly with the cardio‐thoracic ratio decreasing from 0.66 to 0.48, while echo showed a decreased LVDd of 43 mm and an elevated EF of 69%.

## 4 Discussion

Left bundle branch pacing (LBBP) has recently emerged as an innovative approach to correct CLBBB. According to the accordant experiences from several centers, it is a promising way of cardiac resynchronization therapy (Zou et al. 2018; Strauss et al. 2011; Huang et al. 2019; Zhang et al. 2019). In this case, LBBP was successfully performed and the paced QRS morphology presented a selective LBBP pattern under threshold output, which suggested that the pacing site was beyond the blocked area and succeeded in the capture of LBB (Chen et al. 2019). The widened QRS complex due to CLBBB became fully normalized with fusion of the RBB conduction by adjusting the sensed atrial ventricular delay (SAVD). The electro‐synchrony brought along a super‐respondence of CRT in this patient. However, TWOs were found during follow‐up, which in turn led to a decrease in pacing ratio during ventricular tracking mode and affected the efficacy of CRT therapy. From the ECG shown in Figure 3a, we can notice three different patterns of QRS complexes: narrow, intermediate, and widened. The optimal narrow QRS complex (first, fourth and seventh) was the result of successful LBBP, but the following TWO resulted in the pacemaker reaching its upper tracking rate limit and triggered the prolongation of SAVD, which in turn led to an intermediate fusion beat formed by the merging of intrinsic and paced QRS (second, fifth, and eighth). Moreover, the following TWO reset the PVARP, causing the next intrinsic atrial signal to fall into this period and fail to trigger ventricular pacing, which led to the widened baseline CLBBB QRS morphology (third and sixth).

The T‐wave over‐sensing in this case was caused by T‐wave inversion (TWI) during LBBP, which has been observed and reported previously by Geng et al. (2022), and was probably the result of cardiac memory (CM). The idea is that, after the cessation of abnormal ventricular depolarization, the changes in myocardial repolarization can be manifested by a change in the T‐wave axis, also known as electrotonic modulation of the T wave, as a result of the restoration of normal cardiac excitation and the subsequent changes in ventricular repolarizing sequence (Rosenbaum et al. 1982; Walton et al. 2013). Rosenbaum et al. proposed in 1982 that the T waves during normally conducted beats seemed in some way “to remember” the QRS complex of the abnormally conducted beats and showed the same direction as these abnormal QRS complexes, and named this effect ‘cardiac memory’. They confirmed with experiments in their article that the occurrence of left bundle branch block may produce deeply negative T waves (TWIs) during subsequent normalization of conduction, and that this effect exhibits a cumulative nature (the longer LBBB lasted, the longer it took for the TWIs to return back to normal) (Rosenbaum et al. 1982). Detailed mechanism of this phenomenon is still unknown, but has been explored in various animal heart models regarding action potential duration heterogeneity and endogenous ventricular gradients (Walton et al. 2013; Blair et al. 2025). In summary, successful LBBP can alter the ventricular activation sequence of LBBB cases back to normal, resulting in transient and reversible TWIs that may persist for days or even weeks with said mechanism.

Furthermore, despite the fact that the occurrence of prominent T‐wave changes might be the main reason for TWOs, in this case TWOs did not occur immediately after the procedure when TWIs already existed. Hyperkalemia caused by renal failure and side effects of certain medication might be responsible for the high and sharp T‐wave morphology and therefore precipitated the malfunction in this patient, for the sharp T waves with higher amplitude would be more easily identified as R wave by the CRT‐D.

Also, the unique algorithm of ICD might have played a role in this situation as well. With the auto sensing algorithm of ICD, it is more probable for TWOs to happen with the lower sensitivity threshold after ventricular pacing (Brown and Swerdlow 2016). The changing of sensitivity threshold after sensing and pacing also made it more susceptible for CRT‐D to identify the T wave as the R wave.

To the best of our knowledge, this is the first report that describes electrotonic modulation‐related T‐wave over‐sensing after LBBP. The present case suggested that LBBP was capable of correcting the true CLBBB and normalizing the QRS complex completely. However, this significant change in ventricular repolarization sequence could precipitate T‐wave changes in the early phase after pacemaker implantation, which might induce TWOs and interfere with the operation of CRT‐D. This problem can be solved by reprogramming the parameters of the pacemaker. In this case, T‐wave changes improved as time went by, and this problem no longer existed according to our observation. Nevertheless, the phenomenon of electrotonic modulation‐related T‐wave changes after LBBP should receive more attention in future clinical practice.

## Author Contributions

Linlin Li and Manxin Lin: collected and analyzed the clinical data and wrote the manuscript. Xinyi Huang: helped collect and analyze the results of echocardiogram. Jincun Guo and Simei Chen: helped perform CRT‐D programming and analyze the results of electrocardiogram. Fanqi Meng and Binni Cai: recruited the subject, helped collect clinical data and reviewed the manuscript. All authors read and approved the final manuscript.

## Ethics Statement

Informed consent has been obtained from the patient regarding the use of his clinical data for publication.

## Conflicts of Interest

The authors declare no conflicts of interest.

## Data Availability

The data that support the findings of this study are available on request from the corresponding author. The data are not publicly available due to privacy or ethical restrictions.
